# Criterion validity of wrist accelerometry for assessing energy intake via the intake-balance technique

**DOI:** 10.1186/s12966-023-01515-0

**Published:** 2023-09-25

**Authors:** Paul R. Hibbing, Gregory J. Welk, Daniel Ries, Hung-Wen Yeh, Robin P. Shook

**Affiliations:** 1 Department of Kinesiology and Nutrition, University of Illinois Chicago, 1919 W. Taylor St, Rm 650, Mail Code 517, Chicago, IL 60612 USA; 2 Center for Children’s Healthy Lifestyles & Nutrition, Children’s Mercy Kansas City, Kansas City, MO USA; 3 Department of Kinesiology, Iowa State University, Ames, IA USA; 4 Statistical Sciences Department, Sandia National Laboratories, Albuquerque, NM USA; 5 Biostatistics & Epidemiology Core, Children’s Mercy Kansas City, Kansas City, MO USA; 6 School of Medicine, University of Missouri-Kansas City, Kansas City, MO 64108 USA

**Keywords:** Energy balance, Ambulatory assessments, Wearables

## Abstract

**Background:**

Intake-balance assessments measure energy intake (EI) by summing energy expenditure (EE) with concurrent change in energy storage (ΔES). Prior work has not examined the validity of such calculations when EE is estimated via open-source techniques for research-grade accelerometry devices. The purpose of this study was to test the criterion validity of accelerometry-based intake-balance methods for a wrist-worn ActiGraph device.

**Methods:**

Healthy adults (*n* = 24) completed two 14-day measurement periods while wearing an ActiGraph accelerometer on the non-dominant wrist. During each period, criterion values of EI were determined based on ΔES measured by dual X-ray absorptiometry and EE measured by doubly labeled water. A total of 11 prediction methods were tested, 8 derived from the accelerometer and 3 from non-accelerometry methods (e.g., diet recall; included for comparison). Group-level validity was assessed through mean bias, while individual-level validity was assessed through mean absolute error, mean absolute percentage error, and Bland–Altman analysis.

**Results:**

Mean bias for the three best accelerometry-based methods ranged from -167 to 124 kcal/day, versus -104 to 134 kcal/day for the non-accelerometry-based methods. The same three accelerometry-based methods had mean absolute error of 323–362 kcal/day and mean absolute percentage error of 18.1-19.3%, versus 353–464 kcal/day and 19.5-24.4% for the non-accelerometry-based methods. All 11 methods demonstrated systematic bias in the Bland–Altman analysis.

**Conclusions:**

Accelerometry-based intake-balance methods have promise for advancing EI assessment, but ongoing refinement is necessary. We provide an R package to facilitate implementation and refinement of accelerometry-based methods in future research (see paulhibbing.com/IntakeBalance).

**Supplementary Information:**

The online version contains supplementary material available at 10.1186/s12966-023-01515-0.

## Background

Energy intake (EI) plays a key role in regulating body mass [1]. However, accurate measures of EI are difficult to obtain in free-living environments. Self-report instruments are standard tools for this purpose, but they are associated with a high degree of error [2–5], leading to many persistent challenges in dietary research and practice [6–9]. Thus, there is an ongoing need to develop more valid and feasible measures of EI that avoid self-report [10, 11].

The “intake-balance” method is a leading alternative to self-report [12]. This method draws from the principle of energy balance, which is a model of the relationship between energy expenditure (EE), EI, and changes in energy storage (ΔES). The relationship is based on the First Law of Thermodynamics, which states total energy in a system remains constant, although it may be converted from one form to another [13, 14]. When applied to energy balance, the Law dictates that ΔES is negative (i.e., weight loss) when EE exceeds EI, while ΔES is positive (i.e., weight gain) when EI exceeds EE. The nature of this relationship (ΔES = EI – EE) allows any of the variables to be calculated based on the others. Thus, it is possible to back-calculate EI based on observed values for ΔES and EE (i.e., EI = ΔES + EE). Normally, this is done using gold standard methodology for assessing ΔES (repeated scans by dual energy X-ray absorptiometry; DXA) and EE (doubly labeled water; DLW) [14–16]. However, DLW is cost-prohibitive and labor-intensive to use [17]. These factors have led to increased interest in the use of other EE assessment methods within the intake-balance framework [18–21].

Accelerometry is a promising surrogate for DLW [22], but there is currently an evidence gap regarding its use in the intake-balance framework. Preliminary applications have been focused on consumer-grade devices and others for which the manufacturers provide limited information about the prediction algorithms [18–20]. Thus, there is a need to increase the transparency and accessibility of device-based intake-balance assessments. Research-grade devices may be especially useful for this purpose, given the growing emphasis on open-source methodology when using such devices [23–26].

We recently demonstrated proof-of-concept for an open-source and accelerometry-based approach in an interventional setting [27]. However, the study was not designed to test criterion validity. The purpose of the present study is to address that gap by testing the criterion validity of open-source accelerometry methods within the intake-balance framework. A secondary purpose is to compare the validity of these EI estimates to what was achieved by standard assessment techniques (self-report and related tools), as a means of contextualizing the accelerometer-based estimates in comparison to standard practice.

## Methods

### Participants

This is a secondary analysis of data from a prior observational study (clinicaltrials.gov registration number NCT04142281) [20]. Participants were 24 adults who gave written informed consent prior to beginning the study. The procedures were approved by the Children’s Mercy Kansas City Institutional Review Board.

### Protocol

The parent study followed a repeated measures design. Specifically, participants completed two 14-day DLW measurement periods, separated by a 14-day isotope washout period. At the start of each DLW measurement period, participants came to the lab in the morning (before 09:00) after an overnight fast. Their visit included body composition assessment via DXA (Lunar iDXA, GE Healthcare, Chicago, IL, USA) followed by DLW dosing. For the DLW dosing, two urine samples were collected, with 1–2 voids in between. The first sample was collected prior to ingesting the isotopes to determine background isotope abundance. The second was taken 4.5–5.0 h afterward. Participants were then fitted with an ActiGraph GT9X to be worn on the non-dominant wrist for the ensuing 14 days in free living (ActiGraph LLC, Pensacola, FL).

During the two-week free-living assessment, participants provided a third urine sample on Day 7. They also completed 2–3 diet recall surveys in which they reported all food and drink consumed the previous day. As described by Shook et al. [20], the multipass survey methods were carefully designed and consistent with standard practice, including rigorous training for both study staff and participants [28–31]. The surveys were administered by a registered dietician via telephone, using the Nutrient Data System for Research Software, version 2017 [28]. Survey delivery was standardized across participants to ensure consistency and reduce risk for response bias. All surveys were administered on randomly selected non-consecutive days, including at least one weekday and one weekend day.

At the conclusion of the free-living period, participants came back to the lab to return their ActiGraph monitor, provide a fourth urine sample, and have a second DXA scan. The dates and times of all urine samples were logged, and samples were stored in a -80°C freezer until study completion. The samples and logs were then shipped to Pennington Biomedical Research Center (Baton Rouge, LA, USA) for batch analysis in their Mass Spectrometry Core. ActiGraph data were downloaded and stored in raw acceleration format (.gt3x files) and “activity count” format (.agd files, in 60-s epochs).

### Criterion measure of EI

Criterion values for EI were derived by summing EE (DLW) and ΔES (DXA). EE was determined by measuring the isotope elimination rates in the urine samples, which were then used to calculate total EE, expressed as a daily average (kcal/day) [17, 32]. As shown in Eq. 1 [18], ΔES was determined from changes in fat mass (ΔFM, in kg) and fat-free mass (ΔFFM, in kg), with scaling for the duration of the measurement period (i.e., 14 days).

1 $$\begin{array}{c}\Delta ES\;(kcal/day)=\frac{1020\ast\Delta FFM+9500\ast\Delta FM}{14}\end{array}$$

### Comparison measures of EI

A total of 11 methods were tested against the criterion values. Eight were derived from the wrist-worn ActiGraph data, and three were from other techniques. Below, each method is described in greater detail.

#### Accelerometry-based measures

The eight ActiGraph methods were subdivided into four pairs. The first pair included the Hildebrand linear [33, 34] and non-linear [35] methods, both of which were regression-based methods predicting oxygen consumption (VO_2_) from accelerometer data collected at the non-dominant wrist. The calculations were made after combining all three axes of acceleration data (in milli-gravitational units) into a single variable called the Euclidian Norm Minus One (ENMO; Eq. 2). Negative values were rounded up to 0, and second-by-second averages were calculated. The linear method was a piecewise function, as shown in Eq. 3. The non-linear method was a power function, as shown in Eq. 4. Due to the lack of intercept in the non-linear method, a floor value of 3.0 ml/kg/min was applied, consistent with intended use [35]. The same lower bound was applied for the linear method. For both methods, a ceiling of 70 ml/kg/min was applied. Predictions were generated each second for both methods, then smoothed by calculating minute-level averages. Lastly, VO_2_ was converted to kcal assuming a respiratory quotient of 0.85 (4.862 kcal/L from the table of Lusk [36]). The assumed respiratory quotient was chosen due to its prevalence in EE research and the limited amount of accompanying error, relative to individualized values calculated based on dietary intake among weight-stable individuals consuming a western diet [37, 38].

2 $$\begin{array}{c}\mathrm{ENMO}\;\left(milli-g\right)=\sqrt{X^2+Y^2+Z^2}-1\end{array}$$

3 $$\begin{array}{c}VO_2\;(ml/kg/min)=\left\{\begin{array}{c}3.0\;\forall\;ENMO\;\leq\;44.8\\7.28\;+\;0.032\;\ast\;ENMO\;\forall\;ENMO\;>\;44.8\end{array}\right.\end{array}$$

4 $$\begin{array}{c}VO_2\;\left(ml/kg/min\right)=0.901\;\ast\;ENMO^{0.534}\end{array}$$

The second pair of accelerometry-based methods came from Hibbing et al. [39], who presented two-regression methods for the left and right wrists. Both versions were tested in the present study by applying them to the non-dominant wrist data. (The rationale and implications of this approach are discussed later.) Like the Hildebrand methods, the two-regression methods took second-by-second ENMO as input. Predictions were generated in three steps, beginning with application of a sedentary cut point. For non-sedentary observations, a second cut-point was then applied to differentiate continuous walking and running from intermittent activity. The latter cut-point was based on coefficient of variation in the signal, calculated with a specialized sliding window technique described elsewhere [39, 40]. Briefly, the sliding window technique involved calculating the coefficient of variation among each data point and various combinations of its preceding and succeeding data points, then selecting the lowest value. After classifying each non-sedentary data point as either continuous walking and running or intermittent activity, the third step involved predicting EE via activity-specific regression equations (for non-sedentary epochs) or a static EE value of 1.25 METs (sedentary epochs). The left and right wrist methods are summarized in Eqs. 5 and 6, respectively, where CWR, CV, and IA represent continuous walking and running, coefficient of variation, and intermittent activity, respectively. All MET predictions were constrained using floor (1.25 METs) and ceiling (20 METs) limits. Predictions were made for each second of data, then smoothed by calculating minute-level averages. Conversion to kcal was done assuming 1 MET = 3.5 ml/kg/min, then using the same VO_2_ conversion factor described previously for a respiratory quotient of 0.85.

5 $$\begin{array}{c}METs=\left\{\begin{array}{c}Sedentary:\;1.25\;\forall\;ENMO\;\leq\;45.6\\CWR:-12.13+3.1381\ast log\left(ENMO\right)\;\forall\;CV\;\leq\;19.4\%\cap ENMO>45.6\\IA:0.81+0.03033\ast ENMO-0.00005\ast ENMO^2+0.00000002\ast ENMO^3\;\forall\;CV>19.4\%\cap ENMO>45.6\end{array}\right.\end{array}$$

6 $$\begin{array}{c}METs=\left\{\begin{array}{c}Sedentary:\;1.25\;\forall\;ENMO\;\leq\;60.2\\CWR:-8.86+2.6564\ast log\left(ENMO\right)\;\forall\;CV\leq21.2\%\cap ENMO>60.2\\IA:0.82+0.03423\ast ENMO-0.00004\ast ENMO^2+0.00000004\ast ENMO^3\;\forall\;CV>21.2\%\cap ENMO>60.2\end{array}\right.\end{array}$$

The third pair of methods came from Montoye et al. [41], who presented neural networks for the left and right wrists. Like the Hibbing methods, both neural networks were applied to the non-dominant wrist data from the present study. To do this, raw data were summarized every 30 s using percentiles and lagged covariance, which were then fed into the neural networks to predict METs. The values were constrained to a range of 1–20 METs and converted to VO_2_ and kcal in the same manner described previously for the Hibbing two-regression methods.

The final pair of methods came from Staudenmayer et al. [42], who presented a linear regression equation and random forest to predict METs from monitors worn on the dominant wrist. (The applicability of these dominant-specific models to the non-dominant data in this study is discussed later.) Both methods used identical features (*n* = 2) to predict METs every 15 s. The first feature was the standard deviation of the signal vector magnitude, where vector magnitude was the root sum of squares across all three axes. The second feature was the mean inclination angle of the monitor. The linear regression equation is given in Eq. 7. Predictions were treated in the same manner described for the Montoye methods, i.e., by truncating to a range of 1–20 METs, then converting to VO_2_ and finally to kcal.

7 $$\begin{array}{c}METs=1.89378+5.50821\left(SD_{vector\;magnitude}\right)-0.02705\left(mean\;inclination\;angle\right)\end{array}$$

#### Other measures

Three additional EI estimation methods were tested. The first two were obtained from the body weight planner of the National Institute of Diabetes and Digestive and Kidney Diseases (NIDDK) [43]. The estimates were extracted using methods described in our recent interventional proof-of-concept paper [27]. Specifically, we used the online interface (see niddk.nih.gov/bwp) in expert mode with advanced controls activated. We filled in the measured body mass from Days 1 and 14 of each measurement period, along with participant demographics and related information (including physical activity level, based on DLW and predicted basal metabolic rate from Schofield’s equations [44]). The Schofield equations were specific to each participant’s sex and age group, with estimates obtained using weight and height as predictors. Based on these observations and the time elapsed between them, the planner then generated two predictions, one being for weight change (i.e., the predicted daily EI required for accomplishing the observed change in body mass over the course of the measurement period) and the other being for weight maintenance (i.e., the predicted EI required for maintaining the original body mass).

Lastly, we tested self-reported EI from the dietician administered recall surveys. Values were calculated for each participant by taking the mean of their survey responses. This was done separately for each of the two 14-day measurement periods.

### Accelerometer data processing and aggregation

Accelerometer data were screened for non-wear and sleep using the methods of Choi et al. [45] and Tracy et al. [46], respectively. Valid days were defined as having ≥ 10 h of awake wear time, with invalid days (those with < 10 h of awake wear time) being excluded from the analysis. Participant-level screening was also performed, with participants being excluded if they did not have ≥ 4 valid days. On valid days, basal EE values were imputed for minutes that were classified as non-wear or sleep. These values were calculated using Schofield’s equations with weight and height as predictors, again using the specific equations corresponding to each participant’s sex and age group [44]. After calculating total EE for each valid day, an average EE was calculated (kcal/day), which was then summed with ΔES to determine estimates of EI.

### Statistical analysis

Participant characteristics were summarized using mean and SD for continuous variables and frequencies for categorical variables. Excess body fat was summarized using World Health Organization cutoffs of > 25% for males and > 35% for females [47, 48]. Dietary behavior was summarized using the Healthy Eating Index, an instrument that scores diet quality on a scale from 0 to 100 [49, 50].

For each method, we used mixed effects regression to test three accuracy metrics, namely bias (i.e., $predicted-DLW$), absolute error (i.e., $\left|predicted-DLW\right|$), and percentage error (i.e., $\frac{|predicted-DLW|}{DLW}*100\%$). Metrics were first calculated for each participant occasion, then regressed on a null set of predictors with a random participant intercept. The latter formulation allowed the fixed-effect intercepts to reflect a mean value when accounting for repeat testing within participants. Thus, the intercepts reflected mean bias, mean absolute error (MAE), and mean absolute percentage error (MAPE). A total of 33 models were fitted, corresponding to the 3 accuracy metrics applied to 11 measures of EI. *P*-values were adjusted using the false discovery rate correction to account for the number of tests [51].

Error trends were further examined using Bland–Altman methods for repeated measures [52–54]. To do this, we first extracted standard deviation (SD) of the random effects from the aforementioned mean bias models to facilitate calculating limits of agreement ($mean\;bias\pm1.96\ast SD$). We also fitted additional models in which individual bias scores were regressed against criterion values from DLW, represented as a fixed effect. (The DLW values were used instead of the mean of DLW and predictions, because DLW is a criterion measure [55].) A random intercept effect was again included to account for repeat testing within participants. The slope, marginal R^2^, and conditional R^2^ of the resulting models were descriptively examined to assess the degree of systematic error for each method.

Hereafter, summary statistics are given as mean ± SD.

## Results

Table 1 shows participant information. Accelerometer variables, EE values, and EI values are summarized in Table 2. Four male participants had a body fat percentage > 25% (range: 27.3-33.3%), and five female participants had a body fat percentage > 35% (range: 35.0-50.1%). The remaining participants fell in the ranges of 12.8-24.3% (males) and 22.6-34.1% (females). Across all recall assessments, the Healthy Eating Index was 66.5 ± 14.9, considerably higher than the national average of 58 (see https://www.fns.usda.gov/healthy-eating-index-hei).

**Table 1:** Summary of participant characteristics. Values are mean ± SD for continuous variables, and n (%) for categorical variables

	**Female (*n* = 14)**	**Male (*n* = 10)**	**Total (*N* = 24)**
**Age (y)**	29.5 ± 6.1	32.4 ± 10.6	30.7 ± 8.2
**Height (cm)**	168.3 ± 7.8	176.7 ± 4.5	171.8 ± 7.8
**Weight (kg)**	68.7 ± 9.8	80.7 ± 10.7	73.7 ± 11.6
**BMI (kg/m**^**2**^**)**	24.4 ± 4.5	25.8 ± 3.0	25.0 ± 4.0
**Weight Status**			
Healthy Weight	8 (57%)	5 (50%)	13 (54%)
Overweight	4 (29%)	4 (40%)	8 (33%)
Class 1 Obese	2 (14%)	1 (10%)	3 (12%)
**Schofield BMR (kcal/day)**	1,457 ± 112	1,843 ± 134	1,618 ± 228
**Fat Free Mass (kg)**	45.5 ± 5.1	62.3 ± 7.7	52.5 ± 10.4
**Fat Mass (kg)**	23.5 ± 8.8	18.4 ± 7.9	21.4 ± 8.7
**Body Fat (%)**	33.4 ± 8.7	22.4 ± 7.8	28.8 ± 9.9

*BMI* Body mass index, *BMR* Basal metabolic rate

**Table 2:** Summary of accelerometer data, energy expenditure, and energy intake. Values are mean ± SD. *N* = 24, except where otherwise noted

	**First Assessment**	**Second Assessment**	**Both Assessments**
**Sleep Time (h/day)**^a^	8.7 ± 0.9	8.6 ± 1.0	8.6 ± 0.9
**Non-Wear Time (h/day)**	0.2 ± 0.3	0.5 ± 0.7	0.4 ± 0.5
**N Days (DLW/DXA)**	14.0 ± 0.0	14.0 ± 0.2	14.0 ± 0.1
**N Valid Days (accelerometer)**	13.0 ± 0.7	13.1 ± 0.4	13.1 ± 0.6
**ΔEnergy Storage by DXA (kcal/day)**	-145 ± 434	-89 ± 413	-117 ± 420
**Energy Expenditure (kcal/day)**			
DLW	2,524 ± 619	2,474 ± 512	2,499 ± 562
Hildebrand Linear Model	2,207 ± 349	2,188 ± 358	2,197 ± 350
Hildebrand Non-Linear Model	2,346 ± 371	2,318 ± 378	2,332 ± 371
Hibbing Left Wrist 2RM	2,622 ± 415	2,596 ± 416	2,609 ± 412
Hibbing Right Wrist 2RM	2,637 ± 422	2,610 ± 423	2,623 ± 418
Montoye Left Wrist ANN	2,966 ± 454	2,929 ± 446	2,948 ± 445
Montoye Right Wrist ANN	3,089 ± 514	3,045 ± 518	3,067 ± 511
Staudenmayer Linear Model	3,091 ± 483	3,079 ± 490	3,085 ± 481
Staudenmayer Random Forest	3,089 ± 506	3,061 ± 509	3,075 ± 503
**Energy Intake (kcal/day)**			
DLW	2,379 ± 917	2,385 ± 663	2,382 ± 792
Hildebrand Linear Model	2,062 ± 516	2,099 ± 493	2,081 ± 500
Hildebrand Non-Linear Model	2,201 ± 528	2,229 ± 500	2,215 ± 509
Hibbing Left Wrist 2RM	2,477 ± 564	2,507 ± 523	2,492 ± 539
Hibbing Right Wrist 2RM	2,492 ± 567	2,521 ± 525	2,506 ± 541
Montoye Left Wrist ANN	2,821 ± 609	2,840 ± 560	2,831 ± 579
Montoye Right Wrist ANN	2,944 ± 647	2,956 ± 592	2,950 ± 613
Staudenmayer Linear Model	2,946 ± 651	2,990 ± 623	2,968 ± 630
Staudenmayer Random Forest	2,944 ± 645	2,972 ± 607	2,958 ± 620
Body Weight Planner (Weight Loss)	2,556 ± 670	2,416 ± 629	2,486 ± 647
Body Weight Planner (Weight Maintenance)	2,562 ± 579	2,470 ± 524	2,516 ± 548
Self-Report^b^	2,117 ± 538	2,268 ± 626	2,196 ± 583

*DLW* Doubly labeled water, *DXA* Dual energy X-ray absorptiometry, *2RM* Two regression model, *ANN* Artificial neural network

^a^ Calculated as sum of minute-by-minute values for each calendar day (typically with some sleep time in the morning and some in the evening, i.e., not reflective of continuous overnight sleep intervals)

^b^ Calculated after excluding missing participant values from the first and second assessments (*n* = 5 and *n* = 3, respectively)

For five participants, self-report data were incomplete (*n* = 2) or missing altogether (*n* = 3). All available self-report data were used when presenting summary statistics (see Table 2), whereas only the 19 participants with complete data from both timepoints were included when presenting self-report data in the formal analyses. All other results (accelerometry-based and NIDDK) are presented for the full 24-person sample. When using the NIDDK Body Weight Planner, there were three instances where the physical activity level from DLW (i.e., total energy expenditure divided by Schofield predicted BMR) was less than the minimum allowable value in the online system (1.111). The minimum value of 1.111 was used in those cases.

**Fig. 1:**
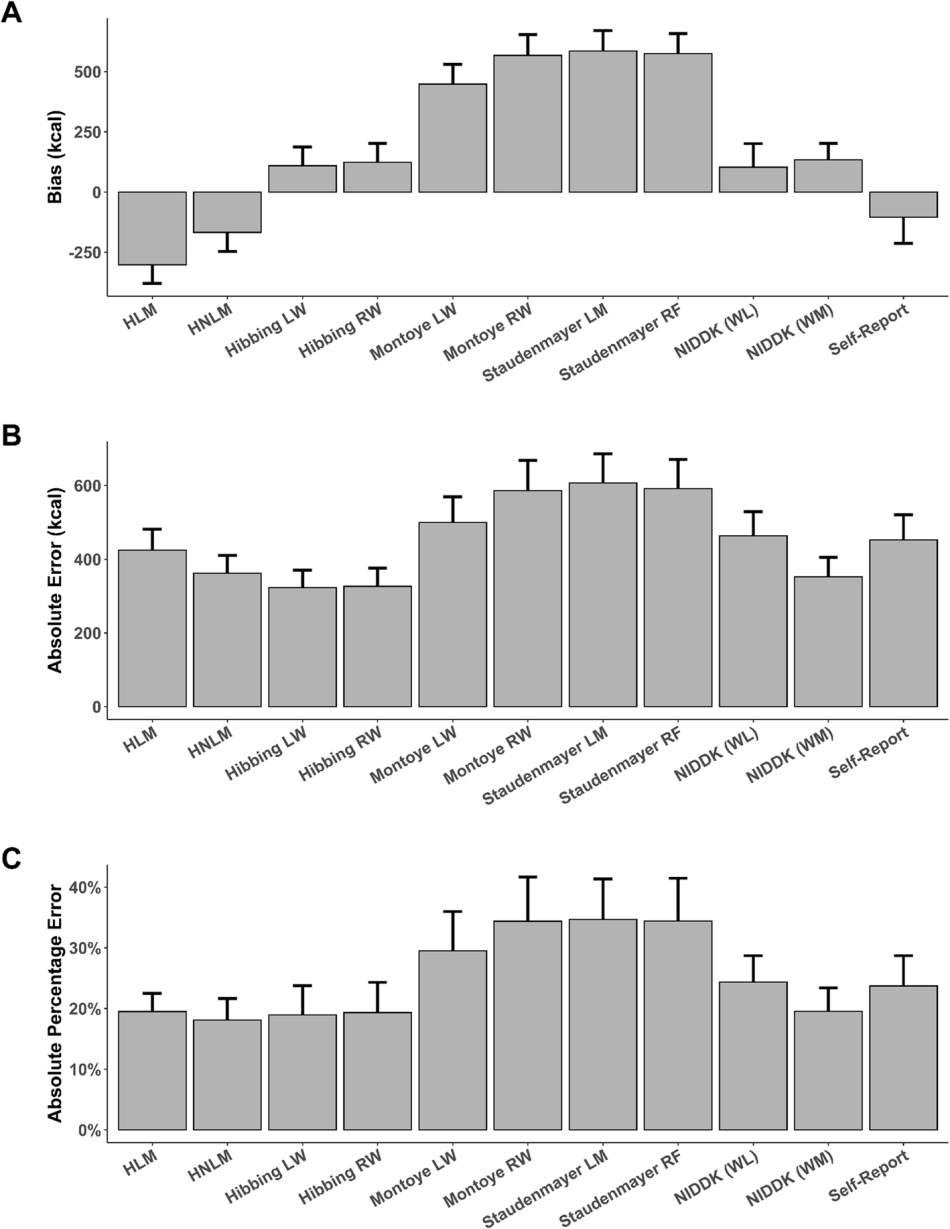
Error metrics for each measure. Values are shown for: **A** mean bias; **B** mean absolute error; and **C** mean absolute percentage error. Error bars are standard error

Figure 1 shows mean bias, MAE, and MAPE. Means and 95% confidence intervals are provided in the supplementary material (see Table S1). The majority of methods tended to overestimate EI, with mean bias ranging from 104 kcal/day (NIDDK weight loss model; *p* = 0.31) to 586 kcal/day (Staudenmayer linear model; *p* < 0.001). In contrast, the Hildebrand and self-report methods tended to underestimate, with mean bias ranging from -302 kcal/day (Hildebrand linear model; *p* < 0.001) to -104 kcal/day (self-report; *p* = 0.35).

Results showed a general distinction between the six best-performing methods (Hildebrand non-linear method, both Hibbing methods, both NIDDK methods, and self-report) and the five remaining methods (Hildebrand linear method, both Montoye methods, and both Staudenmayer methods). Specifically, the distinctions between these groups were fairly consistent when comparing mean bias (± 104–167 for the six best versus ± 301–586 kcal/day for the five others), MAE (323–463 versus 425–607 kcal/day), and MAPE (18.1-24.4% versus 19.5-34.7%). Notably, the NIDDK method for weight loss had the lowest mean bias yet the fifth highest MAE, suggesting the favorable mean bias score was achieved through cancelation of over- and underestimates. In contrast, the NIDDK method for weight maintenance ranked highly for both mean bias and MAE.

**Fig. 2:**
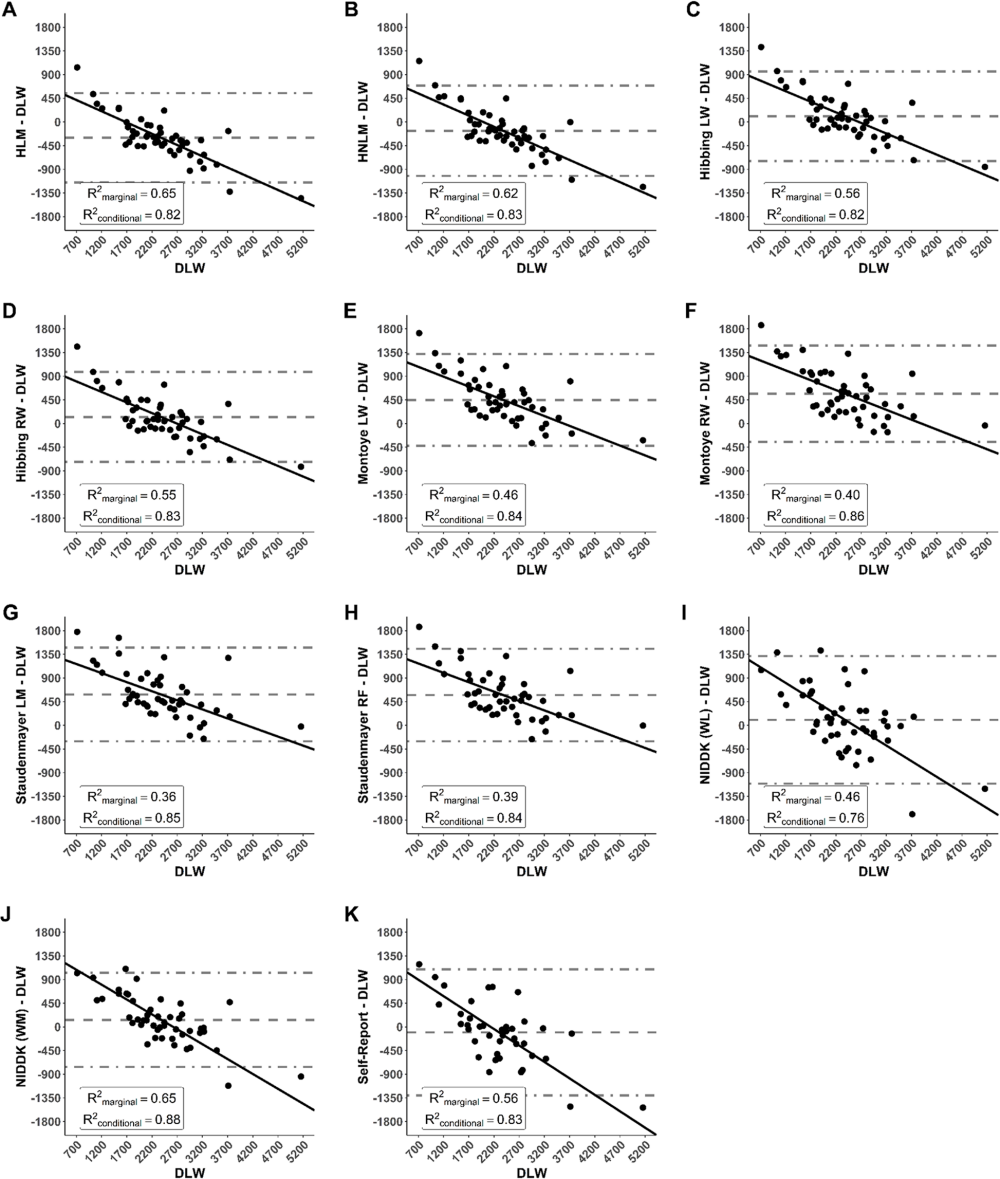
Bland–Altman plots comparing criterion values from doubly labeled water (DLW) against: **A** the Hildebrand linear model (HLM); **B** the Hildebrand non-linear model (HNLM); **C** the Hibbing left wrist (LW) two-regression model; **D** the Hibbing right wrist (RW) two-regression model; **E** the Montoye LW neural network; **F** the Montoye RW neural network; **G** the Staudenmayer linear model (LM); **H** the Staudenmayer random forest (RF); **I** the National Institute of Diabetes and Digestive and Kidney Diseases (NIDDK) body weight planner for weight loss (WL); **J** the NIDDK body weight planner for weight maintenance (WM); **K** self-reported values from dietician-administered recall surveys. Note: The N for self-report was 19 rather than 24, due to exclusion of 5 participants who did not complete surveys at both timepoints

Bland–Altman results are shown in Fig. 2. The standard deviation of bias scores was substantially higher for the NIDDK weight loss and self-report methods (611–619 kcal/day) than for the other methods (434–467 kcal/day). Consequently, limits of agreement were much wider (total widths of 2396–2427 kcal/day versus 1700﻿–1829 kcal/day), indicating worse individual-level validity. Systematic error was evident for all methods, yet in varying degrees. All slopes were negative, with magnitudes of 0.34–0.43 for the accelerometry-based methods versus 0.56–0.63 for the NIDDK and self-report methods. Marginal R^2^ was 0.36–0.46 for the Montoye, Staudenmayer, and NIDDK weight loss methods, versus 0.55–0.65 for the others. In contrast, conditional R^2^ was 0.82–0.88 for all methods except the NIDDK weight loss method (0.76).

## Discussion

### Summary and key findings

In this study, we evaluated the criterion validity of various methods for assessing EI. Our primary focus was the use of accelerometry-based methods for wrist-worn activity monitors, applied within the intake-balance framework. The strongest evidence of criterion validity (both group- and individual-level) was seen for the Hildebrand non-linear method and the two Hibbing methods. It is difficult to fully explain why these methods exceled, but likely factors include the robustness of the original calibration protocols [33, 39] and advantages of the modeling structures themselves (e.g., low susceptibility to overfitting).

A secondary purpose of our study was to compare the validity of accelerometry-based methods to that of prominent non-accelerometry-based methods (i.e., NIDDK and self-report). This allowed examination of the degree to which accelerometry-based methods may improve on the current status quo when measuring EI. The Hildebrand non-linear and Hibbing methods showed promise in this area as well. Specifically, their group-level validity was comparable to the non-accelerometry-based methods, and their individual-level validity was generally better (including substantial advantages over the self-report and NIDDK weight loss methods).

Because the validity of each method in our study was anchored to criterion estimates, the analyses provide valuable insight about the degree of error that can be expected when applying the methods in the field. Taken together, the results suggest wrist-worn accelerometry methods (i.e., the Hildebrand non-linear and Hibbing methods) have competitive validity compared to traditional measures of EI. Below, we discuss the importance of this study and the accelerometry-based intake-balance method, along with sources of error, opportunities for continued development, caveats for interpreting the present findings, and considerations when selecting a method to assess EI in future research.

### Importance of the study and method

To our knowledge, the present study is the first criterion validation of device-based EI estimates when using open-source methodology for a widely used research-grade device (ActiGraph GT9X). This is a step forward, as prior studies have either used closed-source devices [18, 20], or else lacked a criterion measure [27]. The use of open-source methodology is crucial for upholding FAIR principles (Findability, Accessibility, Interoperability, and Reusability) [56, 57] and for combating widespread usability issues in accelerometry [58]. It also provides methodological transparency, in contrast to the well-known “black box” design of most consumer-grade devices [59]. To facilitate ongoing development and application of the accelerometry-based intake-balance methods through open-source channels, we provide an R package and vignette by which the major steps can be automated [60].

The accelerometry-based intake-balance approach offers several key benefits compared to the standard approach with DLW. One of the biggest examples is its relatively low cost, which makes it accessible to a wider range of researchers. A related benefit is that the accelerometry-based method does not require urine collections or isotope analyses, and thus places lower burden on both participants and researchers. Together, these benefits make the accelerometer-based intake balance approach highly scalable for large studies. However, despite its conceptual value and the empirical promise that was shown in this study, there are also important considerations that may require additional research, as discussed below.

### Sources of error and opportunities for refinement

During non-wear periods, the accelerometer-based intake-balance method requires imputation of EE values. For the present study, this was done using estimates of basal metabolic rate from Schofield’s equations [44]. The latter choice was made both for consistency with our original proof-of-concept study [27] and because the Schofield equations remain widely used in accelerometry and physical activity research [61–63]. Nevertheless, other equations (particularly Henry’s [64]) are more common in clinical nutrition research. This represents an opportunity for further testing and refinement of the accelerometer-based intake-balance method, through future studies that test the impact of using different prediction equations. Similarly, our procedures involved an assumed respiratory quotient of 0.85 when converting VO_2_ to kcal. While this is common practice [38] and consistent with our original proof-of-concept study [27], an alternative approach would be to individualize the values by using calculated food quotient in place of the assumed respiratory quotient [37]. Future work could explore how estimates of EI change when using the assumed versus individualized values.

Handedness and sidedness are additional sources of error that may have impacted our results. In this study, participants wore devices on the non-dominant wrist. While this is the most common placement in wrist accelerometry, other placements are also widespread, including placements on a specific side of the body without accounting for dominance [65]. Accordingly, wrist-based equations and models have been developed in different ways, and there is no clear consensus concerning which way is best or how much cross-applicability exists between them. Prior research has frequently shown that EE and physical activity predictions are similar regardless of which wrist the device is worn on [39, 41, 66–69], and thus we chose not to restrict our analysis to methods that were specifically designed for the non-dominant wrist. The appropriateness of this decision was borne out by our results for the Hibbing methods (and, to some degree, the Montoye methods as well), where results were highly similar for the left-sided and right-sided versions. Nevertheless, further comments are warranted on issues of handedness and sidedness.

Both handedness and sidedness have theoretical implications for wrist accelerometry, the former because movement patterns may differ between the dominant and non-dominant wrists [70], and the latter because vertical axis orientation is reversed across wrists [71]. Together with the highly skewed population distribution of handedness [72], this makes it unclear how much measurement error is attributable to handedness versus sidedness. For example, a method that was calibrated for the non-dominant wrist may actually be better suited to the left side (regardless of dominance) unless left-handed individuals were oversampled in the original calibration. Conversely, a method for the right wrist may actually be better suited to the dominant wrist for the same reason.

While it is difficult to conduct a theoretical analysis that untangles the effects of handedness and sidedness on wrist accelerometry, it is easy to perform sensitivity analyses and determine if there are practically significant effects to begin with. This was a key reason for including the Hibbing and Montoye methods in our study, and for testing the left-sided and right-sided versions of each method separately rather than using the left-sided model for right-handed participants and vice versa. As noted above, the results were generally quite similar regardless of which side the models were intended for. This suggests that issues of handedness and sidedness had minimal impact on the data in this study. It may also suggest that none of the methods derived an advantage or disadvantage from the degree of alignment between its original calibration protocol and that of the current study. Nevertheless, these possibilities cannot be fully verified, and our results should be interpreted with commensurate nuance.

When considering the potential for measurement error in this study, it is also important to consider the nature of the protocol itself and the criterion measures. In particular, the present study protocol involved 14-day assessment periods, which were ideal for DLW, yet only long enough to elicit small changes in the DXA measures (FFM and FM). Thus, the precision of DXA is important to consider as a source of measurement error. Prior work has shown the Lunar iDXA to yield rescan reliabilities of 0.5% and 1.0% coefficient of variation for FFM and FM, respectively [73]. Given our sample means of 52.5 kg FFM and 21.4 kg FM, this would translate to potential measurement errors of roughly 0.26 and 0.21 kg, respectively, ultimately propagating to EI errors up to ~ 165 kcal/day (see Eq. 1). Future studies are needed to validate the accelerometer-based intake-balance method over longer time periods, although it should be noted that study duration presents a tradeoff in this respect, with longer protocols being ideal for the assessment of ΔES while shorter protocols are ideal for the assessment of EE.

### Caveats and implications for method selection

While the present findings show promise when using accelerometry-based methods to estimate EI, some caveats are important to consider when interpreting our results and selecting methods for future studies. One important caveat is that our results from self-report and accelerometry-based methods are not directly comparable, due to the differing sample sizes (*n* = 19 for self-report versus 24 for accelerometry) and granularities (2–3 measurements for self-report, versus continuous assessment for accelerometry) of the methods. These factors may influence the level of validity observed in our study. They are also reflective of each method’s strengths and weaknesses, which should be carefully considered when choosing a method in future studies. We have already listed several key benefits of the accelerometry-based approach, with additional strengths including its objectivity and potential for collecting continuous data over extended periods. The key drawbacks of the accelerometry-based approach hinge on managing the large volumes of data collected. Some accelerometry-based methods can also be computationally intensive, leading to lengthy processing time. In contrast, the NIDDK and self-report methods offer convenient and straightforward means of application with a more manageable volume of data. However, they cannot support continuous measurement, nor can they be conveniently automated. That is, self-report requires trained personnel to administer the surveys while the NIDDK method requires manual data entry for each participant, including a module to estimate physical activity level (unless an estimate is provided from another source such as accelerometry). Manual data entry is not only labor-intensive, but can also increase the risk of data entry errors. When selecting a method, further considerations include cost, applicability in different populations such as children and adolescents, and burden on participants and researchers (which may also have implications for quality control).

Our analysis demonstrates another important consideration for method selection, namely that some methods (especially self-report) may perform well at the group level but not the individual level, as evidenced by small mean bias coupled with large MAE and wide limits of agreement. Such methods may be suitable in some situations but not others. For instance, individual-level validity may not be a precondition for studies focused on group comparisons, whereas it is essential for interventions delivering individualized dietary prescriptions. It should also be noted that the present study design did not allow testing sensitivity to change for any of the methods. This makes it unclear which method is most recommendable for research questions focused on change over time. In general, these factors highlight that no single method is the best choice for every study, and selections should be made on a case-by-case basis. However, the present findings provide strong evidence that an accelerometry-based approach can be a valid option in some cases.

### Study strengths and limitations

A strength of the present study was the repeated measures design with criterion measures of EE and ΔES. Few other studies have included these rich characteristics. However, the use of DLW also led to a small overall sample size, which was compounded by missing data for the self-report method. As noted previously, the precision of DXA may have been a source of error in the criterion measurements of ΔES. This limitation could potentially have been addressed by using magnetic resonance imaging instead, although prior work has shown strong agreement between the latter method and DXA when assessing whole-body lean and adipose tissue [74]. Another limitation was that the sample characteristics were not representative of the general population, calling for further research. This includes a need to better understand how the performance of the EI assessment methods may be related with factors such as diet quality and nutritional status.

## Conclusions

Current accelerometry-based intake-balance methods can achieve similar group-level validity to the established NIDDK and self-report methods, along with individual-level validity that is as good or better than the latter methods. The most accurate accelerometry-based methods are the Hildebrand non-linear method and the Hibbing two-regression models. However, all methods showed room for improvement. The accelerometry-based methods can be implemented and refined using the R package developed as part of this study. Future work should examine validity in youth populations and evaluate accelerometry-based methods in terms of sensitivity to change in an intervention setting. Accelerometry-based methods for assessing EI have the potential to increase the accuracy and efficiency of research in nutrition and obesity.

### Supplementary Information

**Additional file 1: Table S1.** Results of mixed effects modeling for estimated energy intake using doubly labeled water and dual energy X-ray absorptiometry as the criterion measures. Values are mean (95% confidence interval).

## Data Availability

Not applicable (secondary analysis).

## Abbreviations

ΔES: Change in energy storage
ΔFFM: Change in fat-free mass
ΔFM: Change in fat mass
DLW: Doubly labeled water
DXA: Dual energy X-ray absorptiometry
EE: Energy expenditure
EI: Energy intake
ENMO: Euclidian norm minus one
MAE: Mean absolute error
MAPE: Mean absolute percentage error
METs: Metabolic equivalents
NIDDK: National Institute of Diabetes and Digestive and Kidney Diseases
SD: Standard deviation
VO_2_: Oxygen consumption
