# Supplementary material for: Criterion validity of wrist accelerometry for assessing energy intake via the intake-balance technique
Source: Int J Behav Nutr Phys Act. 2023 Sep 25;20:115. doi: 10.1186/s12966-023-01515-0 (PMC10521469; doi:10.1186/s12966-023-01515-0)
Supplement: Supplementary file 1 — Additional file 1: Table S1. Results of mixed effects modeling for estimated energy intake using doubly labeled water and dual energy X-ray absorptiometry as the criterion measures. Values are mean (95% confidence interval). [file 12966_2023_1515_MOESM1_ESM.docx]

## SUPPLEMENTARY MATERIAL: Additional Results

## Table S1

**.** Results of mixed effects modeling for estimated energy intake using doubly labeled water as the criterion measure. Values are mean (95% confidence interval).

|  | **Mean Bias** | **MAE** | **MAPE** |
| --- | --- | --- | --- |
| Hildebrand Linear Model | -301.8 (-462.9, -140.7) | 424.9 (308.9, 540.9) | 19.5 (13.3, 25.7) |
| Hildebrand Non-Linear Model | -167.1 (-330.9, -3.3) | 362.2 (263.0, 461.5) | 18.1 (10.8, 25.4) |
| Hibbing Left Wrist 2RM | 109.6 (-52.3, 271.5) | 322.7 (223.7, 421.8) | 18.9 (9.0, 28.9) |
| Hibbing Right Wrist 2RM | 124.0 (-38.6, 286.6) | 326.9 (225.8, 427.9) | 19.3 (9.1, 29.6) |
| Montoye Left Wrist ANN | 448.3 (279.7, 616.8) | 500.0 (357.3, 642.7) | 29.5 (16.2, 42.8) |
| Montoye Right Wrist ANN | 567.7 (390.0, 745.4) | 586.3 (418.0, 754.6) | 34.4 (19.4, 49.4) |
| Staudenmayer Linear Model | 585.8 (411.9, 759.7) | 607.1 (444.9, 769.4) | 34.7 (20.8, 48.5) |
| Staudenmayer Random Forest | 575.6 (405.7, 745.4) | 591.7 (429.3, 754.0) | 34.4 (19.9, 49.0) |
| Body Weight Planner (Weight Loss) | 103.6 (-97.6, 304.7) | 463.5 (327.8, 599.2) | 24.4 (15.4, 33.3) |
| Body Weight Planner (Weight Maintenance) | 133.7 (-6.6, 274.0) | 352.8 (244.0, 461.6) | 19.5 (11.6, 27.5) |
| Self-Report^2^ | -104.3 (-330.4, 121.7) | 453.0 (312.1, 593.8) | 23.7 (13.3, 34.1) |

MAE- mean absolute error; RMSE- root mean squared error; MAPE- mean absolute percentage error; 2RM- two regression model; ANN- artificial neural network.

Note: Dual energy X-ray absorptiometry was the criterion measure of body composition for all intake-balance calculations (doubly labeled water and accelerometry-based methods).
